# Assessment of social vulnerability to floods in the Samin watershed, Indonesia

**DOI:** 10.4102/jamba.v18i1.1947

**Published:** 2026-01-14

**Authors:** Suryanto Suryanto, Sofyan Sholeh, Rahning Utomowati, Agung Hidayat

**Affiliations:** 1 Department of Economics, Faculty of Economics and Business, Universitas Sebelas Maret, Surakarta, Indonesia; 2 Department of Environmental Science, Graduate School, Universitas Sebelas Maret, Surakarta, Indonesia; 3 Department of Geography Education, Faculty of Teacher Training and Education, Universitas Sebelas Maret, Surakarta, Indonesia

**Keywords:** SoVI, flood, socio-economic, PCA, GIS

## Abstract

Floods are a natural hazard that has a major impact on society because of deaths, injuries, property damage, and economic losses. In the context of exposure to flooding, there is a gap between communities or individuals in each region in responding to and dealing with its impacts because of differences in demographic characteristics, regional structure, availability of facilities, and existing disaster prevention and management efforts. In this study, we assessed social vulnerability to flooding in the Samin watershed using the social vulnerability index (SoVI). Social vulnerability index is a quantitative measure that is widely applied to evaluate social vulnerability. This study compiles the stages of indicator selection, data collection, statistical analysis and normalisation, determination of indicator weights and dimensions using principal component analysis, aggregation of indicators, construction of SoVI, and mapping of results. The results show that dimensions related to demographics and exposure are the causes of the majority of social vulnerability variability. Other important dimensions include the socio-economic dimension and growth ratio.

**Contribution:**

Spatial data-based social vulnerability measurement can be used by the government as a basis for formulating flood disaster management policies in the Samin watershed area.

## Introduction

Floods are natural hazards that have a major impact on society because of deaths, injuries, property damage, and economic losses that continue to increase every year (Sung & Liaw 2020; Tanir et al. 2021). However, in the context of exposure to flood disasters, there is a gap between communities or individuals in each region in responding to and dealing with their impacts. These differences are influenced by various social factors, such as demographic characteristics (e.g. the higher vulnerability of elderly populations) (Gu et al. 2018; Sung & Liaw 2020), regional structure (e.g. limited accessibility in smaller regions) (Bucherie et al. 2022a), availability of facilities (e.g. inadequate health and evacuation infrastructure in rural areas) (Lee 2014; Mah et al. 2023), and disaster management efforts (e.g. regular evacuation drills in more prepared regions) (Cong, Feng & Chen 2023).

Through these various factors, differences between regions can be identified by assessing the impact of flood disasters on communities, both in terms of economic losses and lives lost.

Based on the difference in these factors, the use of the social vulnerability index (SoVI) method can analyse each socio-economic characteristic of the community in order to get a clearer picture of the poverty level in each region. Evaluation of social capacity in measuring social vulnerability can determine the potential impact of disasters (Das et al. 2021; Majumder et al. 2023). In fact, the most affected groups of society are often those marginalised by socio-economic inequality (Fatemi et al. 2017; Spielman et al. 2020). This causes social vulnerability to become the basis for assessing the vulnerability of the community as a whole to environmental threats.

Various studies on the measurement of social vulnerability have developed rapidly, both at national and local scales. On a national scale, Fekete (2019) conducted a comprehensive assessment of the implementation of SoVI in Germany and highlighted the challenges of adapting the index to different contexts. Similar applications have been carried out in Norway, which emphasised the role of demographic and economic inequalities (Holand, Lujala & Rod 2011), in China, which revealed spatial disparities in urban resilience (Zhou et al. 2014), in Denmark, which focused on integrating SoVI with climate change adaptation policies (Prall et al. 2024), in Taiwan, which demonstrated its usefulness for flood risk assessment (Sung & Liaw 2020), in the southern United States, which provided one of the earliest empirical validations of SoVI (Emrich & Cutter 2011), in Spain, which stressed the importance of incorporating housing characteristics (Aroca-Jimenez et al. 2017), and in Italy, which highlighted regional differences in vulnerability patterns (Frigerio et al. 2016).

Although these studies provide valuable insights, most of them assume that spatial units such as cities or counties have relatively homogeneous SoVI characteristics. In reality, however, social vulnerabilities within a city can vary considerably across neighbourhoods. Consequently, aggregating SoVI variables at larger spatial units risks overlooking local-scale disparities. This highlights the importance of adopting small-scale, spatially explicit approaches in order to capture variations in social vulnerability more accurately, particularly in urban areas.

Compared with the national scale, attention is now increasingly directed to the study of social vulnerability on a smaller scale, such as cities or districts. Various studies on SoVI at the local scale have been conducted, for example in Tegucigalpa, Honduras (Ebert 2004), which showed large inequalities between informal settlements and formal neighbourhoods; in Bucharest, Romania (Armas & Gavris 2013), which highlighted distinct socio-economic disparities among administrative sectors; in Beijing, China (Zhang & Huang 2013) and Shanghai, China (Gu et al. 2018), which revealed significant variations between urban and peri-urban areas; and in Dhaka, Bangladesh (Masuya 2013), which emphasised the extreme vulnerability of slum communities. These studies clearly indicate that SoVI at the city or district level is not homogeneous but rather shows diverse spatial patterns that require further investigation. However, most of these analyses have not fully adopted a watershed-based approach in SoVI calculations. A watershed-based perspective can provide a more contextual and integrated understanding, particularly in relation to flood disasters, which are strongly influenced by biophysical conditions and spatial planning. Therefore, the main focus of this paper is to design a methodological approach to evaluate aspects of social vulnerability to floods using a watershed-based approach, which can better capture local variations and provide more accurate insights for disaster risk reduction. The watershed approach reflects the overall water flow system from upstream to downstream, so that flood vulnerability analysis becomes more accurate and integrated (Ajtai et al. 2023).

This study emphasises the need for an approach capable of comprehensively examining social vulnerability. One method often used to measure flood vulnerability is through the calculation of a vulnerability index, which is constructed from a number of determining indicators. These indicators describe the condition of a community from various perspectives, including social, demographic, economic, and physical or built environments (Painter et al. 2024; Xiao et al. 2022). These factors play a role in determining the community’s ability to deal with disasters and facilitating the identification of the most vulnerable areas. Therefore, determining the right indicators is a crucial step because it can determine the quality of the analysis results. Indicators also need to be designed according to regional characteristics and the type of threat, while still considering spatial aspects and environmental conditions.

## Research methods and design

### Research area

**FIGURE 1:**
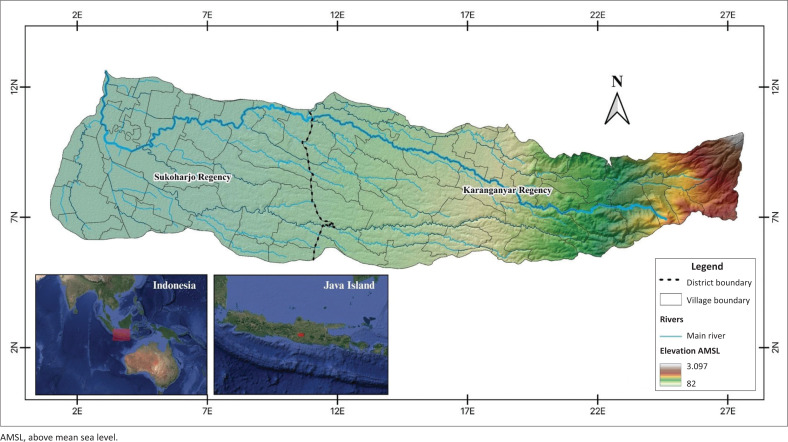
Research area.

The Samin watershed is a watershed that passes through 2 districts, namely Karanganyar Regency and Sukoharjo Regency. It is located at 7° 33′ 57” S - 7° 42′ 54” S and 110° 52′ 27” E - 111° 11′ 27” E (Figure 1).

This watershed has an area of 32,373.46 hectares and a population of 517 602 people with a ratio of 99.57 women to men with a ratio of 99.57 (Statistics Indonesia 2024). Special geographical conditions, such as lowlands and tropical seasonal climates, make the Samin watershed vulnerable to hydrometeorological disasters (Sholeh, Muryani & Suryanto 2025). This watershed is one of the areas experiencing severe land degradation, exceeding its environmental capacity.

On the other hand, the Samin watershed has complex demographic and economic patterns. In the Karanganyar Regency area, there is a livelihood in the field of agriculture and natural tourism, while in Sukoharjo Regency, in the field of industrial services. As a result, it has a heterogeneous regional structure and facilities that can cause SoVI to vary.

### Vulnerability assessment

The analysis of social vulnerability to flooding is carried out at the level of the smallest analysis unit, namely, villages, which are the most basic administrative units in Indonesia. Each village is analysed through an overlay with flood hazard data to identify the level of exposure it is experiencing. In total, 97 villages located within the Samin watershed were identified as the units of analysis. The selection was based on the official administrative boundaries of villages within the watershed (Statistics Indonesia 2024), ensuring that the assessment focuses on the population residing in this area. The SoVI is designed using a measurable and holistic method, with reference to a number of indicators obtained from local data. This kind of index-based approach has been widely used in various studies, such as quality-of-life assessments, land quality evaluations, and social vulnerability analyses to different types of disasters.

The analysis stages in this study consist of several steps, including indicator selection, data collection, statistical analysis and normalisation, and determination of the weight and dimensions of indicators using the principal component analysis (PCA), indicator integration, development of SoVI and visualisation of results in maps. In addition, this study also integrates the exposure dimension, where previous research has confirmed that flood vulnerability is greatly influenced by social factors and geographical exposure conditions.

For example, Ajtai et al. (2023) emphasised demographic factors with flood exposure in the Someș-Tisa River Basin, Romania. Kim and Gim (2020) show a spatial gap in flood exposure between urban and suburban areas. Painter et al. (2024), emphasised the central role of socio-demographic factors in shaping environmental risks.

Meanwhile, Kim, Kang and Hwang (2025) emphasised the importance of integrating the exposure dimension into vulnerability assessments to get a more comprehensive picture of the condition of the society. So that by combining exposures, it can produce a more comprehensive representation of the conditions of vulnerable communities, into a simpler unit, with the contribution of each factor that has been determined through PCA (Ajtai et al. 2023; Aroca-Jiménez, Bodoque & García 2020; El-Zein, Ahmed & Tonmoy 2021). Previous studies have shown that PCA produces empirically stronger findings compared with other approaches (Chakraborty et al. 2020; Cutter 2024). Principal component analysis is done using RStudio software, while spatial processing and result mapping are done using Quantum Geographic Information System (QGIS) software.

**FIGURE 2:**
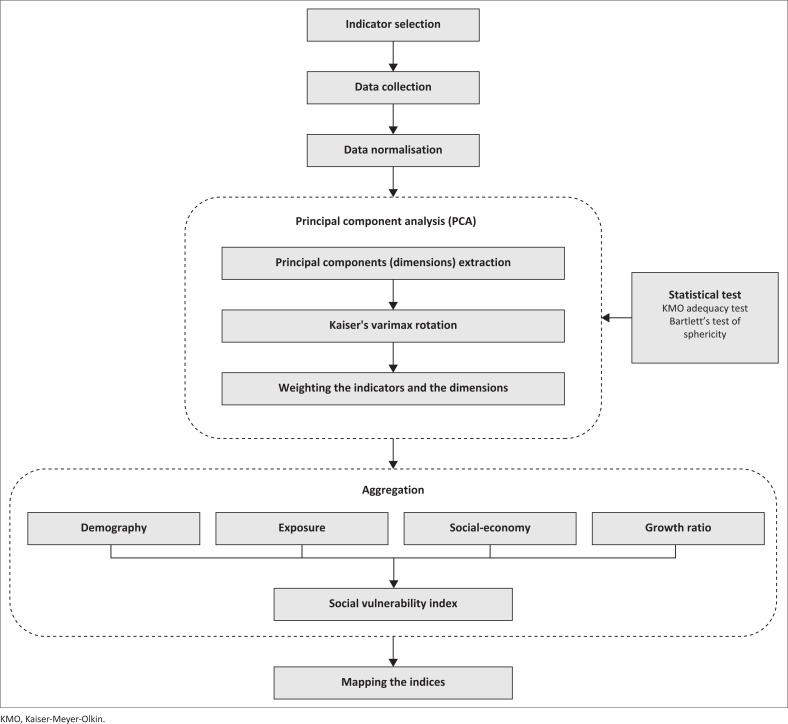
Methodological framework.

Figure 2 shows the methodological framework in this study. The analysis was carried out in several stages, namely, determining indicators, collecting data, normalising data, and implementing PCA. The application of PCA is by extracting the main components (dimensions), the Kaisar varimax rotation and the determination of weights for each indicator and dimension, where the Kaiser-Meyer-Olkin (KMO) method and Bartlett’s Test of Sphericity are the verification of the PCA results. After that, the indicators that have been weighed are combined into a composite index that forms the SoVI in each dimension, namely, demography, exposure, socio-economic, and growth ratio. Furthermore, the extraction sums of squared loadings produce weights that will be used for the multiplication of each dimension. The final result is then visualised through spatial mapping.

### Conceptual framework

The interaction between physical exposure to hazards and the social characteristics of an affected population is widely recognised as a multidimensional construct of vulnerability (Biswas & Nautiyal 2023; Mafi-Gholami, Zenner & Jaafari 2022). Various studies have conducted research related to how communities experience and respond to disasters by showing the composition of demographic conditions, socio-economic conditions, access to resources and environmental characteristics (Ajtai et al. 2023; Aroca-Jiménez et al. 2020; Cutter, Boruff & Shirley 2012; El-Zein et al. 2021).

From a demographic perspective, factors such as age structure, population density and gender composition are empirically the main drivers of vulnerability. The age structure in vulnerable age groups, such as children under 15 years old and adults over 65 years old, causes difficulty in evacuation and recovery (Chen et al. 2021b; Rolfe et al. 2020). Meanwhile, the gender composition where women are larger in number has a very high vulnerability because of different socio-cultural roles and physical access compared with men (Cutter 2024). From a socio-economic perspective, livelihoods in informal sectors such as agriculture, construction and other critical occupations are more vulnerable to economic disruption caused by floods (Bucherie et al. 2022b; Tasnuva et al. 2021).

In addition, environmental and spatial perspectives provide an important perspective on the interaction between exposure and hazard. The increase in built-up areas in flood-prone areas increases exposure to areas that are prone to disasters (Ajtai et al. 2023; Rehman et al. 2019). Thus, hazard parameters such as inundation and flood depth are essential to understand the potential physical and infrastructure damage that results in the long-term socio-economic consequences of flooding (Ajtai et al. 2023; Hinojos et al. 2023). Based on this conceptual foundation, this study developed an integrated framework for the selection of indicators. This framework combines socio-economic, demographic, and environmental dimensions to provide a more holistic representation of societal vulnerability. Based on this approach and taking into account the availability of data, 15 indicators were selected to represent the most relevant determinants of vulnerability in the Samin River watershed.

### Indicator selection

The indicators in this study are based on the context of the local area, relevance and availability of research data. A total of 15 indicators were selected as representational and reflect the demographic, socio-economic and environmental characteristics most relevant to vulnerability analysis in the Samin watershed. The 15 indicators are sourced from the Indonesian Central Statistics Agency (Statistics Indonesia 2024). These characteristics are components that affect the level of disaster exposure and the capacity of the community to respond to flood disasters.

Based on a literature review and the availability of data in the research area, the researcher used indicators of population density, population growth, built – up area and the age factor. Population density indicates the number of individuals in an area that are impacted (Tate et al. 2021). In determining the population density indicator, the population is divided by the administrative area. In addition, the increase in population growth in disaster-prone areas because of mismatches between spatial arrangements increases the social vulnerability of communities (Ajtai et al. 2023; Cutter 2024). This is of particular concern, considering that built-up areas are generally located along rivers, making them more vulnerable to flooding.

The increase in built-up areas contributes to an increase in flood risk because of the increase in watertight areas where water is unable to infiltrate. Indicators that measure the percentage of built-up area and the rate of expansion of built-up areas are integrated into vulnerability analysis (Botezan, Radovici & Ajtai 2022; Rehman et al. 2019). This indicator is calculated based on land cover changes from Landsat-8 satellite imagery in the 2014–2023 period, which was analysed using the Google Earth Engine (GEE) platform. The built-up land class includes areas used for residential and other buildings. The time range was chosen based on the availability of remote sensing data (Sholeh, Suryanto & Saputri 2025).

The age factor is also an important consideration, considering that the elderly and children have limited mobility in emergency situations, thus increasing difficulties in the evacuation process (Chen et al. 2021b; Rolfe et al. 2020; Zhang & Huang 2013). Based on literature studies, this vulnerable group is categorised as residents under 15 years of age and over 65 years old. The proportion of this group is calculated against the total population of the village. In addition, the percentage of women in the overall population in each region is one of the indicators. Women tend to have higher vulnerabilities than men in terms of access to resources, income levels and household responsibilities.

Furthermore, indicators of vulnerable jobs are also considered as one of the aspects of socio-economic vulnerability. Individuals who depend on informal sectors such as agriculture, construction, haircuts, carpentry, sewing, and other menial occupations are considered to be at higher risk of the economic impact of flood disasters (Bucherie et al. 2022b; Tasnuva et al. 2021).

In addition to socio-economic indicators, a number of indicators representing hazard characteristics were also selected. These indicators provide information on the intensity of events, location, and spatial coverage of floods, thus allowing identification of exposed elements and levels of exposure (Ajtai et al. 2023; Hinojos et al. 2023). The extent of the flood area provides an overview of the affected area, while the depth of the waterlogging reflects the potential physical impact on infrastructure and the environment. Even a puddle depth of 0.5 m can cause significant damage to property and pose a major obstacle in the recovery process. In addition to physical damage, floods also have the potential to cause forced displacement, loss of life, and a high burden of recovery costs.

### Data processing and normalisation

The determination of indicators is carried out first, then the values are normalised to obtain a comparable dataset. The normalisation process uses the min-max approach, with a value scale ranging from 0 to 1. Indicators that have high values indicate a greater level of vulnerability, which indicates a positive relationship between the indicator and vulnerability. Conversely, when the value of an indicator increases but causes vulnerability to decrease, this indicates that the indicator is negatively related to the level of vulnerability (see Table 1). Therefore, the normalisation process is applied using Equation 1 for positive relationships and Equation 2 for negative relationships (Gu et al. 2018):

An=(A−Amin)/(Amax−Amin) [Eqn 1]

An=(Amax−A)/(Amax−Amin) [Eqn 2]

Where An is the normalised indicator, *A* is the indicator value, *Amin* is the minimum value of the indicator and *Amax* is the maximum value of the indicator (Table 1).

**TABLE 1:** The principal components extracted.

Component	Initial eigenvalue			Extraction sums of squared loadings		
	Total	% of variance	Cumulative variance %	Total	% of variance	Cumulative variance %
1	4.247	28.312	28.312	4.247	28.312	28.312
2	3.358	22.387	50.699	3.358	22.387	50.699
3	1.993	13.286	63.985	1.993	13.286	63.985
4	1.335	8.899	72.884	1.335	8.899	72.884
5	0.876	5.838	78.722	-	-	-
6	0.788	5.251	83.973	-	-	-
7	0.679	4.526	88.499	-	-	-
8	0.548	3.654	92.153	-	-	-
9	0.302	2.013	94.166	-	-	-
10	0.265	1.765	95.931	-	-	-
11	0.223	1.487	97.418	-	-	-
12	0.165	1.100	98.518	-	-	-
13	0.118	0.787	99.304	-	-	-
14	0.092	0.614	99.919	-	-	-
15	0.012	0.081	100	-	-	-

Note: Only principal components with an eigenvalue greater than 1 were retained for further analysis.

Principal component analysis was then applied to reduce dimensionality and identify the most relevant factors. Based on the eigenvalue > 1 criterion, four principal components were retained, which together explained 72.88% of the total variance (Table 1).

### Weighting using principal component analysis

Determining the weight of each indicator is an important step in vulnerability analysis. Weights reflect how much the role and influence of each indicator is on the SoVI. This decision also has a direct impact on the results of the unit rating analysed. Even so, choosing the most appropriate weighting method is still a problem, because until now there has been no widely accepted standard approach. In practice, many analyses use the same weights or even assign no weights at all to simplify the process and avoid subjective elements and uncertainty. However, this strategy risks ignoring the fact that each indicator has a different degree of influence on social vulnerability, which can reduce the accuracy of the analysis.

The PCA has been widely used for determining the weight of each indicator by previous research, because it is considered more objective than other approaches, such as expert judgement or Participatory mapping (community-based mapping) (Abdrabo et al. 2023; Aflahah et al. 2019; Ajtai et al. 2023; Aroca-Jimenez et al. 2017; Bereitschaft 2020; Bucherie et al. 2022a; Kim et al. 2025; Sung & Liaw 2020; Tasnuva et al. 2021). The PCA technique is performed by extracting components and evaluating the contribution of relative indicators through factor loading.

Furthermore, by performing the Kaiser varimax rotation, the weight of each indicator is calculated based on the value and then normalised to a value of one. Through this method, indicators that have a strong correlation will be greater in covering the same main components (dimensions). Indicators that have a high correlation are consolidated into a single component. The number of extracted components is determined based on an eigenvalue greater than one (> 1), and the interpretability of the results is improved through Kaiser varimax rotation. Next, the weight of each indicator is calculated from the square of the factor loading after rotation, and then scaled so that the total becomes one.

In addition, to obtain the weight of each dimension, the percentage of variance described by each component is divided by the total variance described, as formulated in Equation 3:

DW=(% Varians)/(Total Varians) [Eqn 3]

### Aggregation and development of flood vulnerability indices

**FIGURE 3:**
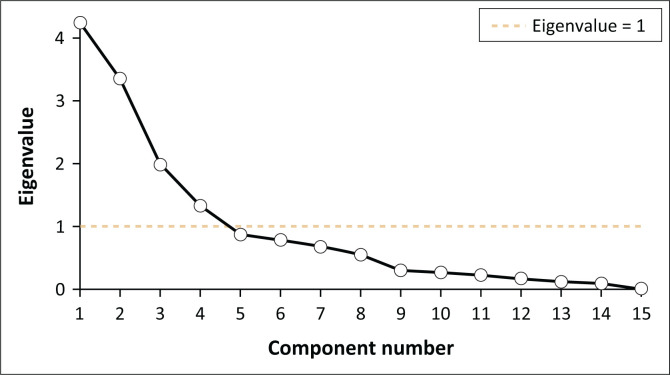
Scree plot.

The indicators are then grouped into four vulnerability indices based on the dimensions obtained through PCA analysis, as shown in Equation 4. Figure 3 presents the scree plot derived from the actual PCA analysis performed on the social vulnerability indicators in this study. This figure is based on real data and not merely conceptual.

Following the Kaiser criterion, which retains components with eigenvalues greater than 1, four principal components were selected for further analysis. These four components have eigenvalues above the threshold and cumulatively explain a significant proportion of the total variance. This decision is also visually supported by the ‘elbow’ in the scree plot, where the curve begins to flatten after the fourth component. Retaining these components allows for a more meaningful aggregation of indicators into four vulnerability dimensions, which are then used to construct the intermediate and final SoVI:

$$\text{Yi}=\sum_{i=1}^{Ni}Xilni$$ [Eqn 4]

In the equation, Yi is the vulnerability index, Xi is the weight of indicator i, and ni is the normalisation value of indicator i.

Social vulnerability index is obtained by multiplying the value of each vulnerability index by the weight of each dimension, then summing up so that the results are obtained. This calculation process is shown in Equation 5:

SoVI=Wd1Yi1+Wd2Yi2+Wd3Yi3 [Eqn 5]

SoVI is classified into five categories (very low, low, medium, high, and very high), with each village assigned to one of the categories based on a vulnerability score calculated using QGIS. This classification process produces clear distinctions between classes while minimising variation within each class. The goal of this method is to simplify internal class differences and highlight contrasts between classes.

### Ethical considerations

Ethical clearance to conduct this study was obtained from the Universitas Sebelas Maret Research Ethics Committee (No. 055/UN27.04/KPPMF/2025).

## Results

### Factor analysis and dimensions

**FIGURE 4:**
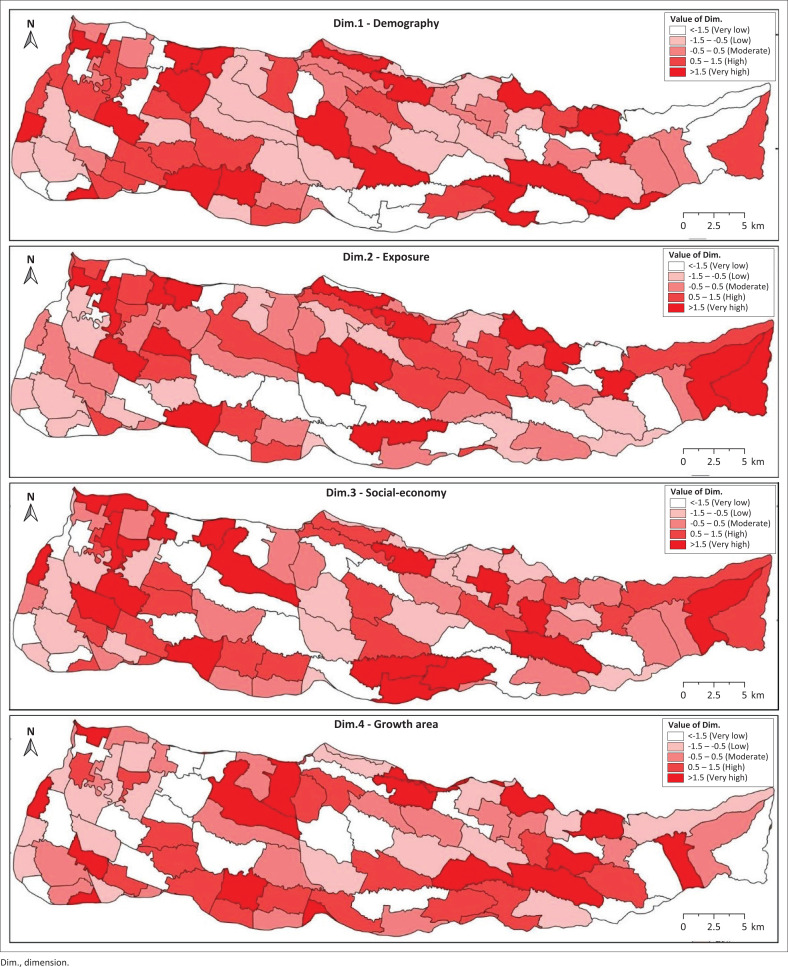
Dimension mapping.

In this study, the application of PCA resulted in four main components extracted based on the criteria of eigenvalue > 1. The four components cumulatively account for 72.8% of the total data variability. The first component contributed 28.31% to the total variance, followed by the second component of 22.38%, the third component of 13.28%, and the fourth component of 8.89% (Table 1). To identify representative indicators on each component, a matrix of components that have undergone a rotation process is used. The indicators with the highest factor loading values in each component are considered to be the dominant reflection of the vulnerability dimension in question. Based on the grouping, each component is analysed and classified into four main dimensions, namely: demographic dimension (PC1), exposure dimension (PC2), socio-economic dimension (PC3), and regional growth dimension (PC4) (Table 2 and Figure 4).

**TABLE 2:** Correlation relationship between 4 components and 15 variables (principal component analysis).

Indicators	Extracted component				Indicator weight	Dimension weight (DW)
	Dim.1	Dim.2	Dim.3	Dim.4		
**Demography (%)**						
Kids (≤ 15 years old)	**0.828**	-	-	-	0.210	0.388
Elders (≥ 65 years old)	**0.911**	-	-	-	0.231	
Vulnerable jobs	**0.913**	-	-	-	0.231	
Low-education	**0.648**	-	-	-	0.164	
Females	**0.647**	-	-	-	0.164	
**Exposure**						
Flood event	-	**0.746**	-	-	0.183	0.307
Flooded area under 0.5 m	-	**0.785**	-	-	0.193	
Flooded areas above 0.5 m	-	**0.785**	-	-	0.193	
Health services	-	**0.785**	-	-	0.193	
Population density	-	**0.493**	-	-	0.121	
Percent of built-up area	-	**0.483**	-	-	0.118	
**Social-economy (%)**						
Poor population	-	-	**0.698**	-	0.544	0.182
Disability	-	-	**0.585**	-	0.456	
**Growth ratio**						
Population growth ratio	-	-	-	**0.384**	0.534	0.122
Built-up growth ratio	-	-	-	**0.334**	0.466	

Note: Bolded numbers show the component extraction results from the PCA statistical calculation.

Dim., dimension.

The demographic dimension consists of the percentage of children aged ≤ 15 years, the elderly ≥ 65 years old, vulnerable workers, people with low levels of education, and the percentage of women. The exposure dimensions include indicators of flood events, the area affected by floods with depths below 0.5 m and above 0.5 m, access to health services, population density, and the percentage of built-up areas. Furthermore, the socio-economic dimension includes indicators of the percentage of poor and disabled people, while the regional growth dimension includes the ratio of population growth and building growth. In the exposure dimension, the flood event indicator, as well as the area of the flood area at a depth of ≤ 0.5 m and ≥ 0.5 m, obtained the highest weight value, which indicates a direct contribution to increasing community vulnerability. In contrast, indicators such as access to health services, population density, and the percentage of built-up areas received lower weights, indicating a relationship that did not directly affect the level of vulnerability in these dimensions.

### Spatial distribution of social vulnerability

The analysis of the selected indicators yielded four main component indices, which were then combined into a SoVI. The calculation involved assigning a weight to each indicator, which was then multiplied by the weight value. Figure 4 shows the contribution of each indicator in forming the vulnerability index. Although the overall variation is not significant, some indicators have a significant influence. For example, communities with high vulnerability, such as the elderly and children, are concentrated in urban areas.

**FIGURE 5:**
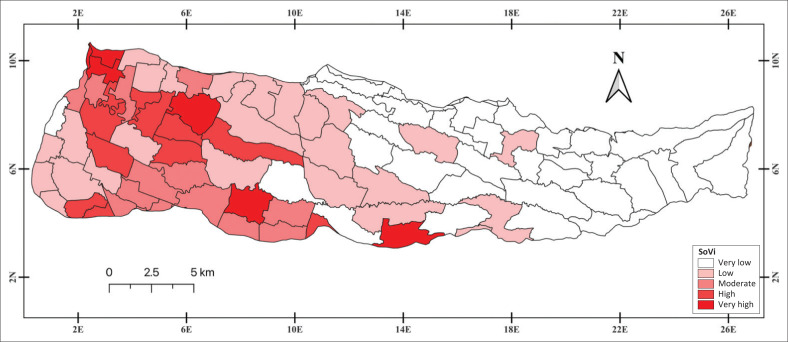
Flood social vulnerability map.

Furthermore, administrative areas located downstream of rivers require special attention because of their higher exposure to flooding. This is reflected in the significant contribution of the floodwater area and water depth indicators. Therefore, Figure 5 plays a strategic role in identifying differences in vulnerability characteristics between regions, thereby providing a more comprehensive understanding of the dynamics of existing social vulnerability.

Figure 4 shows the spatial distribution of each dimension associated with the social vulnerability assessment of the hazard. Lower values are presented in white and higher values in red. The distribution of each dimension spreads.

Areas with high dimension values are composed of flood indicators with depths below 0.5 meters and above 0.5 meters, access to health services, population density, and the percentage of built-up areas and demographic conditions of the community (Dimension 2) with the number of vulnerable people (≤ 15 years and ≥ 65 years), vulnerable workers, residents with low levels of education, and the percentage of women.

The final estimate of the flood disaster in the Samin watershed is calculated by combining four dimensions. The results are mapped at the regional census level and categorised into five main levels for the spatial distribution of the SoVI (Figure 5).

## Discussion

Most of the Samin watershed is explained by the dimensions related to demography (dimension 1), explaining 28.312% of the total SoVI variants in the Samin watershed. This dimension consists of the following variables: Percent of kids (≤ 15 years old), percent of elders (≥ 65 years old), percent of vulnerable jobs, percent of low-education, and percent of females. A percentage measure of the age group – children and the elderly – was associated with a high social vulnerability. This is in line with other studies that show that children and the elderly have a very high vulnerability if exposed to disasters (Guillard-Goncąlves et al. 2015; Tate, Cutter & Berry 2010). However, this is in contrast to other studies that show that the elderly population earns pensions, so they do not have high vulnerability (Goto, Suarez & Ye 2022). In case of the Samin watershed, the majority of people work in the informal sector (farmers, labourers, etc.), where in the elderly, there is no pension money. This is what causes the elderly to have high vulnerability. In addition, the elderly age group faces health problems as a result of ageing (Sun et al. 2017). Regarding low education and the percentage of women, the results show that there are variations. People with higher education are found in urban areas (Chen, Liu, et al., 2021), while the percentage of women is distributed throughout the Samin River watershed.

The dimension related to exposure (dimension 2) accounts for 22.38% of the total SoVI variants in the Samin watershed. This dimension combines several important indicators, including the frequency of flood events, the area affected by floods with depths below 0.5 m and above 0.5 m, access to health services, population density, and the percentage of built-up areas. Areas that experience frequent flooding disasters tend to have high levels of exposure, which directly impacts increased social vulnerability. Low availability and access to health facilities magnify social risks in responding to and recovering from disasters. In addition, the high population density and the large percentage of built-up areas narrow the evacuation space and increase the potential for losses, thus contributing significantly to the high level of social vulnerability in the region.

The socio-economic dimension (dimension 3) contributed 13.286% to the total variation in the SoVI in the Samin watershed. This dimension consists of two main indicators: the percentage of poor people and the percentage of people with disabilities. These two groups are a key focus in social vulnerability studies because of their limitations in coping with and recovering from the impacts of disasters.

Poor communities, for example, face high levels of vulnerability because of limited access to essential resources, information, and services needed both during emergencies and post-disaster recovery. Consistent with previous research findings (Mason et al. 2021; Peek & Stough 2010), people with disabilities also face physical, social, and informational barriers that can slow responses to early warnings and complicate evacuation and aid access. Limited mobility and dependence on others further increase their vulnerability to disaster risks.

Meanwhile, the dimension related to the growth ratio (dimension 4) contributed 8.89% to the total SoVI variation in the Samin watershed. This dimension includes two main indicators: population growth ratio and building growth ratio. The interaction between population growth and the growth of physical settlements illustrates the socio-economic and environmental dynamics in disaster risk reduction. Rapid population growth increases the pressure on a region’s infrastructure capacity in dealing with disasters. In addition, the expansion of buildings that are unbalanced in accordance with the spatial layout where the area is a flood-prone area will increase the vulnerability of the area. This is in line with previous research (Bouaakkaz, Zine El Abidine & Lhoussaine 2023; Tate et al. 2021), which asserts that population growth and settlement expansion can increase social vulnerability because of population concentration in certain areas.

## Conclusion

This research produced an analysis of social vulnerability to flooding in the Samin Watershed, which has variations and differences in each administrative area. The differences between individuals and communities in each region in responding to and overcoming the impact of disasters are influenced by demographic, exposure, socio-economic and growth ratio dimensions. The analysis shows that demographic and exposure dimensions contribute the most to variations in SoVIs, while socio-economic dimensions and growth rates also play an important role in shaping people’s vulnerability levels.

This study fills the knowledge gap with respect to the spatial patterns of social vulnerability in the Samin watershed. By analysing the spatial distribution of SoVI in the Samin watershed at the smallest level, we found that social vulnerability is not homogeneous in the city. The western region has a higher vulnerability to flooding because of vulnerable employment and low education in areas with regular flood events. Furthermore, data on social vulnerability can be used as a basis for spatial planning to control urban expansion into floodplain areas and minimise factors that increase exposure. The approach used is able to uncover more deeply the factors that cause vulnerability in the research area, while providing a more comprehensive perspective for flood risk management authorities in formulating appropriate mitigation and adaptation strategies.
